# Prevalence and Molecular Characterization of *Mycoplasma* Species, *Pasteurella multocida*, and *Staphylococcus aureus* Isolated from Calves with Respiratory Manifestations

**DOI:** 10.3390/ani12030312

**Published:** 2022-01-27

**Authors:** Youserya M. Hashem, Walid S. Mousa, Eman E. Abdeen, Hanaa M. Abdelkhalek, Mohammed Nooruzzaman, Ahmad El-Askary, Khadiga A. Ismail, Ayman M. Megahed, Ahmed Abdeen, Enas A. Soliman, Gamal Wareth

**Affiliations:** 1 Mycoplasma Department, Animal Health Research Institute, Agricultural Research Center, Dokki, Giza 12618, Egypt; Yousreya@ahri.gov.eg; 2 Department of Animal Medicine and Infectious Diseases, Faculty of Veterinary Medicine, University of Sadat City, Sadat City 32897, Egypt; 3 Department of Bacteriology, Mycology, and Immunology, Faculty of Veterinary Medicine, University of Sadat City, Sadat City 32897, Egypt; eman.abdeen@vet.usc.edu.eg; 4 Buffaloes Diseases Research Department, Animal Health Research Institute, Agricultural Research Center, Dokki, Giza 12618, Egypt; hwahba929@gmail.com; 5 Department of Pathology, Faculty of Veterinary Science, Bangladesh Agricultural University, Mymensingh 2202, Bangladesh; mohammed.nooruzzaman@bau.edu.bd; 6 Department of Clinical Laboratory Sciences, College of Applied Medical Sciences, Taif University, P.O. Box 11099, Taif 21944, Saudi Arabia; ahmedelaskary@azhar.edu.eg (A.E.-A.); khadigaah.aa@tu.edu.sa (K.A.I.); 7 Department of Veterinary Public Health, Faculty of Veterinary Medicine, Zagazig University, Zagazig 44159, Egypt; ammegahed@vet.zu.edu.eg; 8 Department of Forensic Medicine and Toxicology, Faculty of Veterinary Medicine, Benha University, Toukh 13736, Egypt; ahmed.abdeen@fvtm.bu.edu.eg; 9 Center of Excellence in Screening of Environmental Contaminants, Benha University, Toukh 13736, Egypt; 10 Department of Bacteriology, Mycology, and Immunology, Faculty of Veterinary Medicine, Benha University, Toukh 13736, Egypt; enas.soliman@fvtm.bu.edu.eg (E.A.S.); gamal.wareth@fli.de (G.W.); 11 Friedrich-Loeffler-Institut, Institute of Bacterial Infections and Zoonoses, 07743 Jena, Germany

**Keywords:** respiratory diseases, *Mycoplasma*, *Pasteurella*, *Staphylococcus*, PCR, sequencing

## Abstract

**Simple Summary:**

Respiratory infection is one of the most common problems facing the livestock industry in Egypt, and results in substantial economic losses. Several bacterial pathogens are implicated in respiratory infections in young calves. In our study, *Mycoplasma bovis*, *Mycoplasma bovigenitalium*, *Pasteurella multocida*, and *Staphylococcus aureus* were the most common bacterial pathogens isolated from calves suffering from respiratory manifestations in Menoufiya Governorate, Egypt. However, the results revealed a low prevalence of these pathogens compared to other studies carried out in Egypt. This may be due to the low number of samples or application of vaccination programs in the study area. Mixed infection is the main perceived criteria in this study. The genetic characterization of these pathogens revealed the high identity and similarities within several global and local linages, proposing the probability of disseminating these bacterial pathogens through several routes during animal contact and movements and animal trading between different geographic locations around the world.

**Abstract:**

Bovine respiratory disease (BRD) is a complex syndrome associated with high mortality in young calves and causes severe economic losses in the cattle industry worldwide. The current study investigated the prevalence and molecular characterization of common bacterial pathogens associated with respiratory symptoms in young calves from Sadat City, one of the largest industrial cities in Menoufiya Governorate, Egypt. In between December 2020 and March 2021, 200 mixed-breed young calves of 6–12 months were examined clinically. Of them, sixty (30%) calves showed signs of respiratory manifestations, such as coughing, serous to mucopurulent nasal discharges, fever, and abnormal lung sound. Deep nasal (Nasopharyngeal) swabs were collected from the affected calves for bacteriological investigation. Phenotypic characterization and identification revealed *Mycoplasma bovis*, *Mycoplasma bovigenitalium*, *Pasteurella multocida*, and *Staphylococcus aureus* in 8.33%, 5%, 5%, and 5% of the tested samples, respectively. The PCR technique using species-specific primer sets successfully amplified the target bacterial DNA in all culture-positive samples, confirming the identity of the isolated bacterial species. Partial gene sequencing of *16S rRNA* gene of *M. bovigenitalium*, *P. multocida*, and *S. aureus*, and *mb-mp 81* gene of *M. bovis* revealed high nucleotide similarity and genetic relationship with respective bacterial species reported from Egypt and around the world, suggesting transmission of these bacterial species between animal host species and localities. Our study highlights the four important bacterial strains associated with respiratory disorders in calves and suggests the possible spread of these bacterial pathogens across animal species and different geographic locations. Further studies using WGS and a large number of isolates are required to investigate the realistic lineage of Egyptian isolates and globally.

## 1. Introduction

Bovine respiratory disease (BRD) is one of the major problems in the livestock industry [1,2]. BRD causes poor health, high mortalities among young calves, reduced carcass weight, impaired animal welfare, and increases treatment and vaccination costs in affected herds [3]. Increased levels of morbidity and mortality in infected calf herds require massive use of antimicrobial compounds, leading to the emergence of antimicrobial resistance [4]. The pathogenesis of BRD is complex and often involves interactions of infectious agents, environmental and stress factors, and the host immune responses [5]. Bovine herpesvirus type 1, bovine parainfluenza-3, bovine viral diarrhea virus, and bovine respiratory syncytial virus are the most reported viral pathogens associated with respiratory diseases in calves [6]. At the same time, many pathogenic bacteria, such as *Pasteurella multocida*, *Mannheimia haemolytica*, *Mycoplasma bovis*, and other opportunistic bacteria, such as *Streptococcus pneumoniae*, *Staphylococcus aureus*, *Pseudomonas aeruginosa*, *Corynebacteria*, *Haemophilus*, *Escherichia coli*, and *Klebsiella pneumonia* are involved in respiratory syndromes in calves [2,7,8]. Most of these bacterial species are commensal in the upper respiratory tract of cattle. Stresses or viral infections lower the immune responses of the animal and pave the way for opportunistic bacteria to colonize the respiratory system, be inhaled into the lungs, and lead to bronchopneumonia [9].

*P. multocida* is one of the opportunistic pathogens associated with BRD under stress conditions, causing high mortality and expenses [10,11]. Similarly, *M. bovis* emerged as a worldwide distributed pathogen causing bronchopneumonia, mastitis, arthritis, and gynecological syndromes in cattle herds [12,13]. Many studies in Egypt reported *M. bovis* as a crucial agent associated with respiratory infection and abortion in the cattle population [14,15]. Although *S. aureus* is the main bacteria associated with bovine subclinical mastitis [16], methicillin-susceptible *S. aureus* and methicillin-resistant *S. aureus* (MRSA) are considered potential pathogens involved in calf pneumonia, as well as a potential risk element for human transmission [10]. The ultimate diagnosis of most bacterial diseases relies principally on isolation and identification through classical bacteriological techniques. However, modern genotypic characterization can provide reliable techniques for rapidly detecting various bacterial organisms [2]. Furthermore, specific studies employing sequencing techniques and targeting specific genes are crucial to identifying infectious bacterial pathogens [17].

In Egypt, the total number of cows in 2015 was approximately 4,883,196 heads, and only 402,070 dairy cows were reared in sizeable commercial dairy farms, while most cattle populations are scattered in the form of smallholders [18]. In addition, the steady increase in the population of Egypt and, consequently, the high increase in consumption of meat and milk demand a constant increase in animal production. The government has also developed plans to increase commercial dairy and beef farming in the new cities near the desert all over the country. However, outbreaks of diseases, particularly respiratory infections considerably dented the success of cattle farming in Egypt [14,19,20,21,22]. As respiratory infections incur severe economic losses and fatal complications in young calves due to pneumonia, this study aimed to determine the prevalence, molecular characterization, and sequencing analysis of the most common bacterial pathogens associated with the respiratory manifestations in young calves in Sadat City, one of the most important cities targeted for reclamation and animal production sectors by the government in Egypt.

## 2. Materials and Methods

### 2.1. Ethics Statement

This study was performed according to the rules and regulations of the Faculty of Veterinary Medicine, the University of Sadat City, Egypt (Approval no. VUSC-014-2-20). This study followed the guidelines of the ethics committee and current legislation on research and ethical approval of the Faculty of Veterinary Medicine (Local ethical approval), University of Sadat City, Egypt.

### 2.2. Study Area

The current study was conducted in Sadat City of the Menoufiya Governorate, one of the largest industrial cities in Egypt. The city is located between 30.3811° N and 30.5266° E, and about 94 km northwest of Cairo, and it is a first-generation new urban community in the country. The majority of residents in the Menoufiya Governorate live in rural areas, with an urbanization rate of only 20.6% [23]. The city is considered one of the most critical areas where the government focuses on agriculture and animal husbandry. Therefore, animal breeding constitutes the main occupation for a majority of the inhabitants.

### 2.3. Animals and Samples Collection

Two hundred mixed breed calves (6–12 months old) from a feedlot cattle farm in Sadat City were clinically examined during the period from December 2020 to March 2021 using repeated visits to the farm. Only 30% (*n* = 60) of examined animals showing respiratory manifestations, including coughing, nasal discharges, abnormal lung sound, with or without fever, were sampled. Deep nasal swabs (nasopharyngeal) were collected from the diseased calves (*n* = 60) using sterile swabs and were transported in a transport medium. Different transport mediums were used. The nutrient broth was used to isolate *S. aureus*, PPLO broth was used to isolate *Mycoplasma*, and trypticase soy broth was used to isolate *P. multocida.* All swabs were transported under cooling conditions, in a Coleman cool box containing ice, to the laboratory for bacteriological examination.

### 2.4. Phenotypic Isolation and Identification of Mycoplasma spp., S. aureus, and P. multocida

For *Mycoplasma* isolation, all nasopharyngeal swabs were cultured on pleuropneumonia-like organism (PPLO) broth (Oxoid Ltd., Basingstoke, UK) at 37 °C for 3–7 days, then on PPLO agar medium (Oxoid Ltd., Basingstoke, UK) at 37 °C for 14 days with 5% CO_2_. Samples with no visible growth within the first week of inoculation were incubated until 21 days before being considered as *Mycoplasma* negative. The typical “fried egg” colonies were developed, and after that, selected *Mycoplasma* colonies were transferred into PPLO broth medium and incubated at 37 °C for 3–7 days. The classical biochemical tests, e.g., glucose fermentation test, tetrazolium reduction test in aerobic condition with 5% CO_2_, film and spot, and arginine deamination tests were used to identify and characterize *Mycoplasma* spp., as described previously [24].

At the same time, nasopharyngeal swabs were spread on Baird–Parker agar (Oxoid Ltd., Basingstoke, UK) and sheep blood agar (Oxoid Ltd., Basingstoke, UK), and then incubated at 37 °C for 24–48 h for isolation of *Staphylococci*. Gram staining, catalase, and coagulase tests were used to confirm *Staphylococcus aureus*, as described previously [25]. The virulence activities of *Staphylococcus aureus*, such as hemolytic activity and DNase agar testing, were performed, as described earlier [26]. In addition, biofilm activity on Congo red medium was tested, as previously described [27]. Isolation of *P. multocida* was performed by streaking the nasopharyngeal swabs onto a selective medium, tryptose blood agar base (Difco BD, Franklin Lakes, NJ, USA), and incubated overnight at 37 °C under aerobic conditions for 48 h. Based on colony morphology, the presumption *P. multocida* colonies were sub-cultured and identified based on morphological and biochemical characteristics described previously [28].

### 2.5. Molecular Confirmation of Mycoplasma spp., S. aureus, and P. multocida Strains by PCR

Genomic DNA extraction was carried out using three typical phenotypic colonies of fresh bacterial culture of the obtained bacterial strains (*Mycoplasma* spp., *S. aureus*, and *P. multocida*) using a Gene JET Genomic DNA purification Kit (Thermo-Scientific, Waltham, MA, USA) following the manufacturer’s instructions. DNA concentration was determined with a spectrophotometer at a 260/230 nm wavelength. PCR was used to confirm *P. multocida* and *Mycoplasma* species, and to confirm *S. aureus* isolates through detection of the *nuc* and *coa* genes. Primers used in the current study are listed in Table 1.

A 50 µL reaction volume was prepared containing 25 µL 2× PCR Master Mix (DreamTaq Green PCR Master Mix, ThermoScientific, Waltham, MA, USA), 1 µL (10 pmol/µL) of each primer, 2 µL (50 ng/µL) DNA and rest of the volume was adjusted to 50 µL with deionized water. The PCR reaction was carried out in a gradient thermal cycler (S1000 Thermal cycler Bio-RAD, Hercules, CA, USA). The standard thermal profile recommended by the kit manufacturer was followed during PCR. The annealing temperature mentioned in the table above was used for each primer set. The PCR products (15 µL) were analyzed by agarose gel (1.5%) electrophoresis and visualized under UV light in a gel documentation system.

### 2.6. Sequencing and Phylogenetic Analysis

Sanger dideoxy sequencing was applied for the partial gene sequencing of the *16S rRNA* gene of *M. bovigenitalium*, *P. multocida*, and *S. aureus*, and the *mb-mp 81* gene of *M. bovis*. The PCR products of the four selected bacterial species (*M. bovis*, *M. bovigenitalium*, *S. aureus*, and two *P. multocida* isolates), showing clear bands, were purified using the GeneJET PCR Purification kit (Thermo Scientific, Waltham, MA, USA). The purified PCR products were sequenced by GATC Biotech Company in Konstanz, Germany, using forward and reverse primers. Homogeneity analysis of the nucleotide and amino acid sequence of our studied isolates and related global strains was performed using BLAST 2.0 and PSI-BLAST search programs (http://www.ncbi.nlm.nih.gov/, date accessed: 20 March 2021), respectively. Multiple alignments with reference strains and the deduction of amino acid sequences were performed using BioEdit [36] and MegAlign software (DNASTAR, Lasergene^®^, Version 7.1.0, Madison, WI, USA). A neighbor-joining phylogenetic tree was built using MegAlign software. A random bootstrapping value of 111 was applied.

### 2.7. Data Submission

The partial nucleotide sequences of the sequenced genes, *mb-mp 81* and *16S rRNA,* from the *M. bovis*, *M. bovigenitalium*, two *P. multocida* isolates, and *S. aureus* were submitted to GenBank with the accession numbers MZ234705.1 *M. bovis*/1; MZ066722.1 *M*. *bovigenitalium* strain bg1; PM-S-2-YWE-EG020, PM-S-3-YWE-EG020, and staph-YWE-EG020, respectively.

## 3. Results

### 3.1. Prevalence of M. bovis, M. bovigenitalium, P. multocida, and S. aureus Recovered from Calves with Respiratory Signs

During a period of four months, from December 2020 to March 2021, 60 (30%) out of 200 calves in a feedlot cattle farm were examined. The clinical examinations revealed signs of respiratory manifestations, including coughing, serous to mucopurulent nasal discharges, depression, and abnormal lung sound with fever in most cases. Bacteriological examination of the nasopharyngeal swab samples (*n* = 60) from the calves with respiratory manifestations was carried out, and confirmation and differentiation were performed using a series of biochemical tests (Supplementary Material Table S1). *M. bovis* and *M. bovigenitalium* were differentiated using glucose fermentation, arginine hydrolysis, tetrazolium reduction test, and film and spot assay, and then confirmed by molecular identification by gene-specific PCR. The *S. aureus* was biochemically tested using catalase, coagulase, DNase tests, and biofilm activity on Congo red medium followed by molecular detection of the *coa* and *nuc* genes. Finally, based on the positive biochemical activity of catalase, oxidase, indole, and nitrate reduction tests, *P. multocida* was confirmed. The *P. multocida* isolates showed negative reaction to Voges–Proskauer, citrate reduction, and urease tests. Sugar fermentation tests revealed that *P. multocida* isolates were positive for glucose, fructose, galactose, sucrose, maltose, and mannose and negative for lactose and salicin (Supplementary Material Table S1).

The proportionate distribution of four bacterial species isolated from calves with respiratory diseases is shown in Table 2. Results of culturing of 60 nasopharyngeal swabs collected from calves with respiratory signs in this study are shown in Supplementary Material Table S2. Among the four different pathogens, *M. bovis* has been isolated from five cases (8.33%), while each of *M. bovigenitalium*, *P. multocida*, and *S. aureus* was isolated from three cases (5%).

Two cases showed mixed infection by *M. bovis*, *M. bovigenitalium*, and *S. aureus*, and another two cases showed mixed infection by *M. bovis* and *P. multocida.* The mixed infection with *P. multocida* and *S. aureus* was seen in one case, and mixed infection with *M. bovis* and *M. bovigenitalium* was seen only in one case. All the tested samples showed mixed infections, as shown in Supplementary Material Table S2.

### 3.2. Molecular Detection of M. bovis, M. bovigenitalium, P. multocida, and S. aureus Isolated from Calves with Respiratory Signs

PCR was employed for molecular detection of the obtained bacterial species using standard and unique primer sets (Table 1). The PCR method successfully amplified the target genes in all isolated bacteria (Supplementary Material Figures S1 and S2). Firstly, a common primer sets targeting the *16S rRNA* gene of class Mollicutes was employed, which amplified a 580 bp fragment in nine *Mycoplasma* positive cultures. Further species-specific PCR detected *M. bovis* isolates at 447 bp fragment in five cases, while *M. bovigenitalium* was amplified at 321 bp in three cases. On the other hand, *P. multocida* isolates were confirmed at 460 bp in three cases. In addition, the PCR amplified a 1318 bp fragment of *16S rRNA* gene and *coa* gene at 987 bp in three *S. aureus* isolates.

### 3.3. Sequence Analysis of M. bovis, M. bovigenitalium, P. multocida, and S. aureus Isolated from Calves with Respiratory Signs

**Figure 1:**
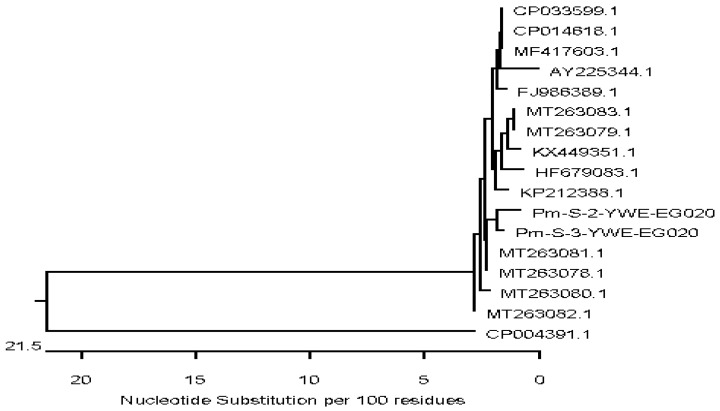
Neighbor-joining phylogenetic tree showing the genetic relationship of two *P. multocida* strains isolated from respiratory samples of calves and global strains. Two *P. multocida* strains with accession numbers PM-S-2-YWE-EG020 and PM-S-3-YWE-EG020 were generated in this study.

The *16S rRNA* gene was used for sequencing of *M. bovigenitalium*, *P. multocida*, and *S. aureus*, and the *mb-mp* 81 gene was used for sequencing of *M. bovis*, then analyses of the *16S rRNA* and *mb-mp* 81 were performed. Partial gene sequencing of representative isolates of each bacterial species was performed due to limited resources. The sequences were subjected to phylogenetic analysis to assess the genetic similarity with local or global strains. The *M. bovis* isolates showed a high nucleotide sequence similarity with two *M. bovis* strains isolated from bovine lung tissues and joint from Canada with accession numbers CP069057 and CP022593, and with five more *M. bovis* isolates from cattle in Belgium with accession numbers CP058503, CP058464, CP058514, CP0558473, and CP058463. Sequence analysis of the *M. bovigenitalium* isolate revealed a high nucleotide similarity with many *M. bovigenitalium* isolates from various sources and countries ranging between 95% and 100%. These include one strain from a bovine vaginal swab in Japan (AP017902), two isolates from cattle in Germany (AY121097 and AY121098), and one isolate from the United States (CP069057). High nucleotide sequence similarity was also found with one Egyptian *M. bovigenitalium* isolate recovered from bovine nasal swab, and one isolate each from vaginal swab and lung tissues of sheep (HQ661815 and MK789488) from South Africa and Turkey, respectively. Additionally, a high similarity in the study of *M. bovigenitalium* isolates with two isolates from the bovine genital tract in Japan and United Kingdom (LC158833 and LR214970) was also found. Sequencing of the two *P. multocida* strains in our study revealed a high nucleotide similarity (97.1–98.7%) and was located in the same subcluster with two *P. multocida* isolated from camel tracheal and nasal samples in Egypt (MT263081, MT263078), two strains from bovine lung tissues in China (CP033599.1, CP014618.1) and one isolate from sheep in India (MF417603) (Figure 1).

**Figure 2:**
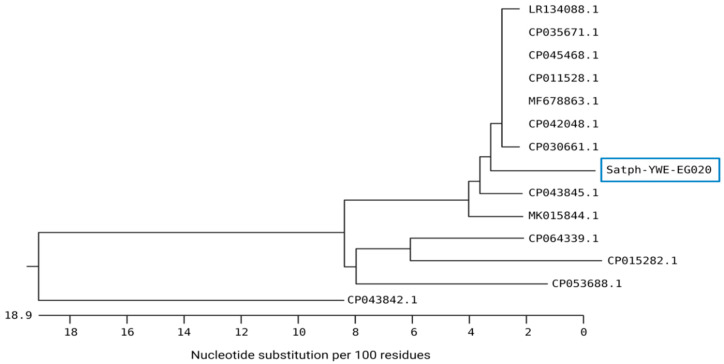
Neighbor-joining phylogenetic tree showing the genetic relationship of *S. aureus* strains isolated from respiratory samples of calves and global strains. *S. aureus* strain with accession number staph-YWE-EG020 was generated in this study.

In the same context, the sequence analysis of *S. aureus* YWE-EG020 generated in the current study revealed a high nucleotide similarity with several *S. aureus* isolates from various sources and localities (Figure 2). Our isolate was located in the same subcluster, with two identical isolates from the USA (CP042048 and CP030661) isolated from retail and human blood specimen samples and shared 96.8–96.9% genetic similarities, respectively. High identity (96.9%) was also noticed for four *S. aureus* strains from China (CP045468), Germany (CP011528), India (CP035671), and the United Kingdom (LR134088). Moreover, a high similarity of the study isolate was also noticed with other *Staphylococcus* species, such as *S. epidermidis* from Korea (CP043845) with a 95.2% identity and *S. pseudintermedius* (MK015844) with a 94.8% identity.

## 4. Discussion

Bovine respiratory disease (BRD) is a multifactorial illness in calves that produces significant mortality and economic losses in cattle farming [3,37]. The outbreak of BRD involves environmental and management stress, infectious agents, and their interactions [5]. Under stress, various commensal bacteria in the upper respiratory tract disturb the local immune defense mechanisms and significantly damage the lungs, and fatal diseases usually occur [9]. In this study, 200 young calves were examined, of them, 60 (30%) showed respiratory signs. The appearance of respiratory manifestations may be as a result of bacterial, viral, or parasitic infection. The bacteriological analysis detected mixed infection by *M. bovis*, *M*. *bovigenitalium*, and *S. aureus* in two cases, as well as mixed infection by *M. bovis* and *P. multocida* in the other two cases. Meanwhile, mixed infection between *M. bovis* and *M. bovigenitalium* occurred in only one case. Finally, the mixed infection with *P. multocida* and *S. aureus* was found in one case among the positive samples collected from calves suffering from respiratory manifestations. The prevalences detected in the present study were higher than in a previous study that isolated *P. multocida* from nasopharyngeal swabs and lung tissue of 15.25% pneumonic calves in Ethiopia [38]. A bacteriological study of respiratory affections in calves in Egypt reported 18.2% *P. multocida*, among which 4.9% were single infection while the rest of the cases had co-infection with *S. aureus*, *E. coli*, and *Streptococcus* sp. in six different combinations [11]. In our study, all the positive bacteriological samples showed co-infection. On the other hand, *Mycoplasma* spp. is associated with a high incidence of BRD in calves, as shown in a Brazilian cohort that reported a 7.4% prevalence rate [1]. However, the study reported *Bacillus* sp., *Staphylococcus intermedius*, and non-fermentative Gram-negative bacteria as the most prevalent bacteria isolated from the lower respiratory tract of calves with respiratory diseases, although these pathogens were not targeted in the present study [1]. A recent study in Egypt examined 225 calves; of these, 55 were apparently healthy and 170 were diseased, and it was found that *E. coli* (16.4%) and *S. aureus* (10.9%) were the most prevalent bacteria in apparently healthy calves; while *E. coli* (23.5%), *Proteus vulgaris* (12.4%) and *S. aureus* (11.8%) were most prevalent in pneumonic calves [2]. A previous study also reported *M. haemolytica* as the main, and a serious, bacterial agent associated with calf pneumonia with other bacteria, such as *P. multocida*, *Histophilus somni*, and *M. bovis*, and mixed infections usually occurring [39]. In Pennsylvania, Soehnlen and colleagues studied respiratory samples of 252 random calves from four veal herds collected at an abattoir and reported *M. bovis* alone, *P. multocida* alone, *M. haemolytica* alone, *M. bovis* and *P. multocida* co-infection, and *M. bovis* and *M. haemolytica* co-infection in 41.1%, 1.1%, 1.1%, 7.8%, and 4.4% of samples, respectively [40]. Besides bacterial pathogens, a virus such as bovine viral diarrhea virus (BVDV) is also involved with pneumonia in feedlot calves, and high co-infection rates with *M. bovis*, *M. haemolytica*, *P. multocida*, and *S. aureus* have been detected [41]. The wide variation between studies in the prevalence rates of the bacteria associated with calf pneumonia may be attributed to the differences in the geographic locality, herd size, animal breed, time of sampling, management factors, stresses, and the common prevalent bacteria in the study areas. *Mycoplasma* infection is a serious problem in small ruminants in Egypt, among both healthy animals or animals with respiratory signs [42]. *P. multocida* and *M. haemolytica* are the most common pathogens causing pneumonia in calves in upper Egypt, reaching 87–100% in some farms [20]. The mixed infection of both *P. multocida* and *M. haemolytica* with *S. aureus* was prevalent among calves in several farms in Upper and Middle Egypt [21]. Additionally, the low prevalence of these bacterial species may be related to the low number of animals included in this study, and/or due to application of a regular vaccination programs against respiratory pathogens. Two national vaccine products produced by the Veterinary Serum and Vaccine Research Institute (VSVRI) against the main causes of BRD were used nationwide and in the study area. Pneumo-bac is used to protect calves from *P. multocida* and *M. haemolytica*, while the Pneumo-4 vaccine is used to protect calves from viral respiratory diseases, e.g., BVD type 2 and 2 virus and IBR, as well as para influenza and syncytial virus.

PCR assays provide a specific and rapid detection of different bacterial pathogens associated with cattle pneumonia compared to the traditional isolation procedure [43]. In our study, we confirmed the diagnosis of isolated bacterial species by PCR, using unique primer sets for the isolated bacteria. All of the isolated bacteria showed positive reactions in species-specific PCR techniques. For *Mycoplasma* detection, PCR was applied using the universal primer sets targeting the *16S rRNA* gene specific to the Mollicutes class. Further serotyping of the *Mycoplasma* spp. into *M. bovis* and *M. bovigenitalium* was done using the *Mb-mp* and *Mbg* gene specific primers, respectively. Similarly, specific primer sets targeting the *kmt1* and *sau* genes were used for the molecular detection of *P. multocida* and *S. aureus*. Our study corroborates several studies in Egypt that employed PCR for rapid detection of the bacterial species recovered from respiratory manifestation in young calves. For example, Abed and colleagues applied PCR as a confirmatory test to identify *P. multocida* using a specific universal gene (*kmt1*) from pneumonic calves in Upper Egypt [20]. A recent study recommended PCR as the principal molecular technique for detecting *Mycoplasma* spp. and *M. bovis* in pneumonic calves [44].

The sequence analysis for four bacterial species was applied to assess their genetic similarity with various local or global strains. The results showed a high genetic relatedness of the sequenced gene of *Mycoplasma* spp. in our study (*M. bovis* and *M.* *bovigenitalium*) with several clones from local and international linages. This was in line with an earlier study that reported that the *M. bovis* ST10 linage was the most prevalent clone in most China provinces, especially in the Ningxia Hui Autonomous region [45]. Another phylogenetic study in Belgium based on gene sequences of 100 *M. bovis* isolated from dairy, beef, and veal farms during five years period reported five major clusters and one outlier of *M. bovis* where the Belgian isolates clustered with Israeli, European, and American strains [46]. In Egypt, sequence analysis of *gapA* and *uvrC* genes of *M. bovis* isolates from mastitic cattle and buffaloes revealed the conserved nature of the *gapA* gene, while amino acid substitutions were found in *uvrC* genes among cattle and buffaloes [47].

The sequencing of two *P. multocida* isolates from the current study shared high nucleotide similarities with strains reported from cattle and other animal species in Egypt and other countries. Hassan et al. demonstrated a close relationship between *P. multocida* isolates from buffalo and cattle rather than between cattle and sheep, and the sequencing of *P. multocida* by *16S rRNA* and *kmt1* genes have been suggested as an important tool in selecting the antigen responsible for protection within the same group and thereby aid in the development of new *Pasteurella* vaccines [48]. Comparative sequence analysis of the *16S rRNA* gene has been suggested as a basic tool to investigate the genetic diversity and reclassification of *P. multocida* across animal species [49]. Moreover, the *16S rRNA* gene sequencing of five isolates of *P. multocida* serotype B: 2 from buffalo, cattle, pig, sheep, and goat indicated a close relationship of hemorrhagic septicemia causing *P. multocida* serotype B: 2 isolates of buffalo and cattle with other uncommon hosts (pig, sheep, and goat) [50].

Several Egyptian and global studies performed the comparative sequence analysis of *S. aureus* isolated from bovine subclinical mastitis and various food sources [16,51,52,53]. A previous study employing the *16S rRNA* genes sequencing and microarray analysis revealed six clonal complexes of *S. aureus* recovered from household cattle and buffaloes in Egypt [54]. Analysis of the *mecA* gene sequences of *S. aureus* obtained from different food products and human pus samples reported high nucleotide similarities between the *S. aureus* strains from different origins [53]. Another genotyping assay using the Staphylococcal protein A (*spa*) gene sequences of *S. aureus* strains isolated from cattle and camels in Egypt revealed the presence of eight SPA types; the most common SPA type was t359 that found in five isolates of *S. aureus* [55].

## 5. Conclusions

*M. bovis*, *M. bovigenitalium*, *P. multocida*, and *S. aureus* were isolated from young calves with respiratory signs and were confirmed by PCR. The presence of such pathogens in calves’ herds threatens the livestock industry and causes a significant public health problem for farmers and workers. Partial gene sequencing and phylogenic analysis revealed a relationship between the Egyptian isolates and many global strains. Thus, emphasizing the role of the transmission of the bacterial strains through several routes during animal contact as well as movements and trade may be possible. In the next-generation sequencing (NGS) technology era, whole-genome sequencing (WGS) can analyze the entire genomes and provide high-resolution in-depth information and high throughput data to track disease outbreaks. Further studies are required for more epidemiological surveillance to help design appropriate control and preventive measures of respiratory diseases in calves.

## Figures and Tables

**Table 1:** PCR primers for molecular detection and characterization of *M. bovis*, *M. bovigenitalium*, *S. aureus*, and *P. multocida* isolates.

Organism	Gene	Primer Name	Primer Sequence (5′-3′)	Anneal. Temp.	Amplicon Size	References
Class Mollicutes	*16S rRNA*	MW28-F MW29-R	CCAGACTCCTACGGGAGGCA TGCGAGCATACTACTCAGGC	55 °C	580 bp	[29]
*M. bovis*	*mb-mp 81*	Mb-mp 81 F Mb-mp 81 R	TATTGGATCAACTGCTGGAT AGATGCTCCACTTATCTTAG	54 °C	447 bp	[30]
*M. bovigenitalium*	*16S rRNA*	Mbg F Mbg R	CGTAGATGCCGCATGGCATTTACGG CATTCAATATAGTGGCATTTCCTAC	60 °C	321 bp	[31]
*P. multocida*	*16S rRNA*	KMT1T7 KMT1SP6	GCTGTAAACGAACTCGCCAC ATCCGCTATTTACCCAGTGG	64 °C	460 bp	[32]
*S. aureus*	*16S rRNA*	Sau 327 Sau 1645	GGA CGA CAT TAG ACG AAT CA CGG GCA CCT ATT TTC TAT CT	55 °C	1318 bp	[33]
*S. aureus*	*coa*	G2 G3	ACCACAAGGTACTGAATCAACG TGCTTTCGATTGTTCGATGC	55 °C	987 bp	[34]
*S. aureus*	*nuc*	*Nuc*	GCGATTGATGGTGATACGGTT AGCCAAGCCTTGGAACTAAAGC	55 °C	270 bp	[35]

**Table 2:** Prevalence of *M. bovis*, *M. bovigenitalium*, *S. aureus*, and *P. multocida* isolated from calves with respiratory signs.

No. of Calves Examined	No. of Calves Showing Respiratory Signs		*M. bovis*		*M. bovigenitalium*		*S. aureus*		*P. multocida*	
	No.	%	No.	%	No.	%	No.	%	No.	%
200	60	30	5	8.33	3	5	3	5	3	5

% was estimated according to the total positive samples (60).

## Data Availability

All authors agree that the data presented in this study are openly available through the MDPI platform or others without any restrictions. The partial nucleotide sequences of the strains from the *M. bovis*, *M. bovigenitalium*, *P. multocida*, and *S. aureus* were submitted to the GenBank with the accession numbers MZ234705.1 M. bovis/1; MZ066722.1 M. bovigenitalium strain bg1; PM-S-2-YWE-EG020, PM-S-3-YWE-EG020, and staph-YWE-EG020, respectively.

## Supplementary Materials

The following are available online at https://www.mdpi.com/article/10.3390/ani12030312/s1, Figure S1: Molecular detection of *Mycoplasma* spp. by PCR. Agarose gel images showing (a) amplification of 580 bp fragment of the *16S rRNA* gene of bacterial species belonging to the class Mollicutes, (b) amplification of 447 bp fragment of the *mb-mp 81* gene of *M. bovis* isolates, and (c) amplification of 321 bp fragment of the *16S rRNA* gene of *M. bovigenitalium* isolates. Lane M: 100 bp DNA ladder, Lane c+ve: Positive control, Lane c–ve: Negative control, Lane 1–4: samples. Figure S2: Molecular detection of *P. multocida* and *S. aureus* by PCR. Agarose gel images showing (a) amplification of 460 bp fragment of the *16S rRNA* gene of *P. multocida*, Lane 1: 100 bp DNA ladder, Lane 2: Positive control, Lane 3: Negative control, Lane 4–6: samples. (b) amplification of 1318 bp fragment of the *16S rRNA* gene of *S. aureus* isolates. Lane 1: 100 bp DNA ladder, Lane 2: Positive control, Lane 3: Negative control, Lane 4–7: samples. Table S1. Biochemical identification of *M. bovis*, *M. bovigenitalium*, *S. aureus*, and *P. multocida* recovered from calves with pneumonic signs. Table S2. Culture and PCR results of 60 nasal swabs collected from pneumonic calves in this study.
