# Supplementary material for: Prevalence and Molecular Characterization of Mycoplasma Species, Pasteurella multocida, and Staphylococcus aureus Isolated from Calves with Respiratory Manifestations
Source: Animals (Basel). 2022 Jan 27;12(3):312. doi: 10.3390/ani12030312 (PMC8833736; doi:10.3390/ani12030312)
Supplement: Supplementary file 1 [file animals-12-00312-s001.zip › animals-1494276-supplementary.pdf]

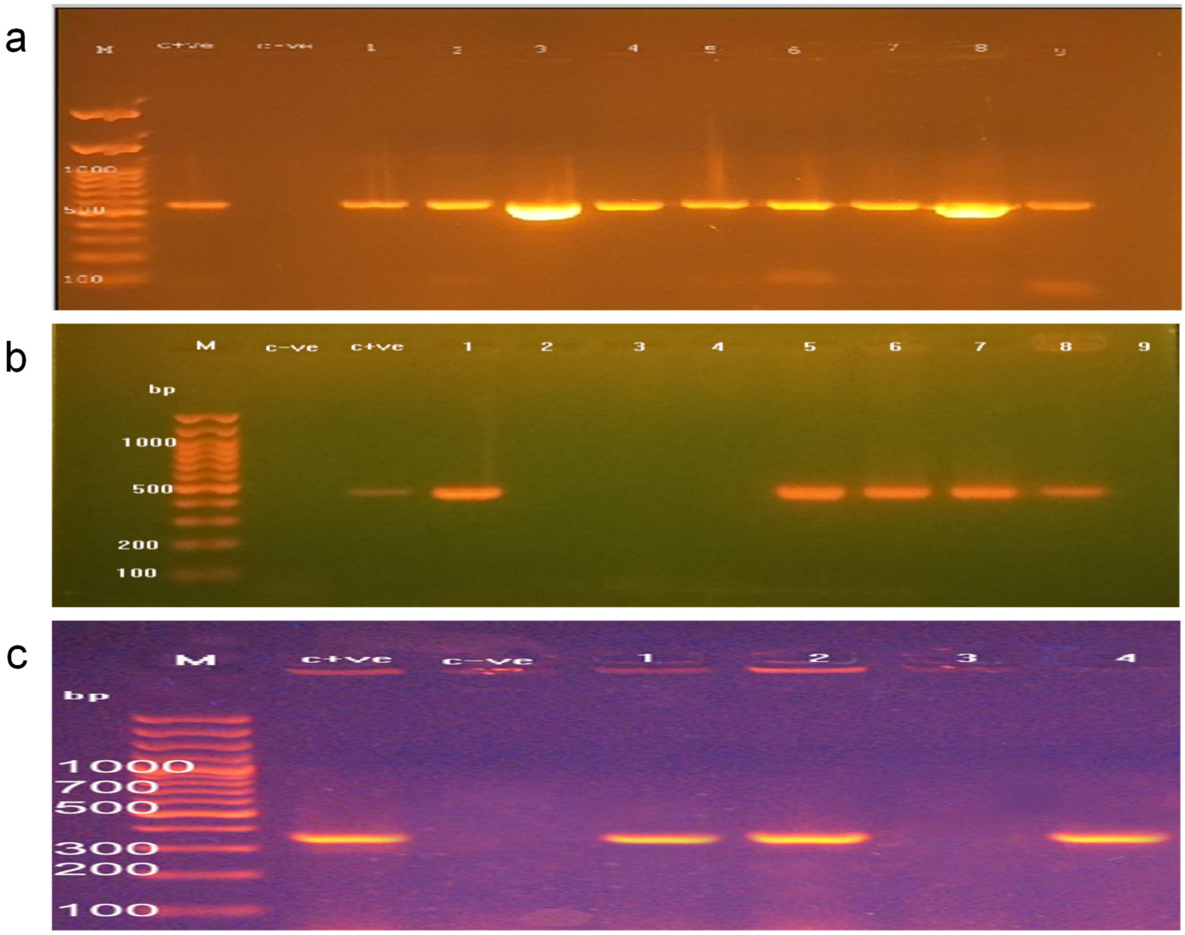

Figure S1. Molecular detection of *Mycoplasma* spp. by PCR. Agarose gel images showing (a) amplification of 580 bp fragment of the *16S rRNA* gene of bacterial species belonging to the class Mollicutes, (b) amplification of 447 bp fragment of the *mb-mp 81* gene of *M. bovis* isolates, and (c) amplification of 321 bp fragment of the *16S rRNA* gene of *M. bovigenitalium* isolates. Lane M: 100 bp DNA ladder, Lane c +ve: Positive control, Lane c –ve: Negative control, Lane 1-4: samples.

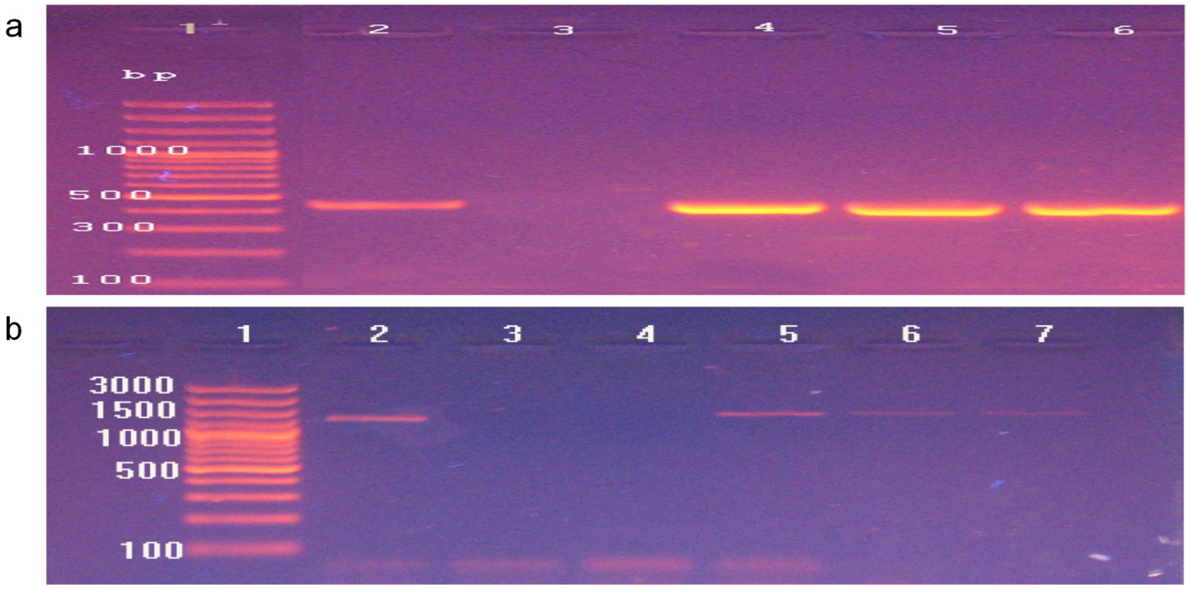

Figure. S2. Molecular detection of *P. multocida* and *S. aureus* by PCR. Agarose gel images showing (a) amplification of 460 bp fragment of the *16S rRNA* gene of *P. multocida*, Lane 1: 100 bp DNA ladder, Lane 2: Positive control, Lane 3: Negative control, Lane 4-6: samples. (b) amplification of 1318 bp fragment of the *16S rRNA* gene of *S. aureus* isolates. Lane 1: 100 bp DNA ladder, Lane 2: Positive control, Lane 3: Negative control, Lane 4-7: samples.

Table S1. Biochemical identification of *M. bovis*, *M. bovigenitalium*, *S. aureus,* and *P. multocida* recovered from calves with pneumonic signs.

Pasteurella multocida															
Catalase	Oxidase	Indole	Nitrate reduction	Urease	Citrate	VP	Gelatin liquefaction	Sugar fermentation tests							
								Glu	Fru	Gal	Suc	Mal	Man	Lac	Sal
+	+	+	+	-	-	-	-	+	+	+	+	+	+	-	-

Staphylococcus aureus				*Mycoplasma bovis*				*Mycoplasma bovigenitalium*			
Catalase	Coagulase	DNase	Congo red	Film & spot	Glu	Arg	TRT	Film & spot	Glu	TRT	Arg
+	+	+	+	+	-	-	+	+	-	+	-

Note: VP: Voges-Proskauer, Glu: Glucose, Fru: Fructose, Gal: Galactose, Suc: Sucrose, Mal: Maltose, Man: Mannose, Lac: Lactose, Sal: Salicin, Arg: Arginine, TRT: tetrazolium reduction test.

Table S2. Culture and PCR results of 60 nasal swabs collected from pneumonic calves in this study.

	M. bovis		*M. bovigenetalium*		*P. multocida*		*S. aureus*	
Sample ID	Culture	PCR	Culture	PCR	Culture	PCR	Culture	PCR
1	-	-	-	-	-	-	-	-
2	-	-	-	-	-	-	-	-
3	-	-	-	-	-	-	-	-
4	-	-	-	-	-	-	-	-
5	-	-	-	-	-	-	-	-
6	P	P	P	P	-	-	P	P
7	-	-	-	-	-	-	-	-
8	-	-	-	-	-	-	-	-
9	-	-	-	-	-	-	-	-
10	-	-	-	-	-	-	-	-
11	P	P	P	P	-	-	P	P
12	-	-	-	-	-	-	-	-
13	-	-	-	-	-	-	-	-
14	-	-	-	-	-	-	-	-
15	-	-	-	-	-	-	-	-
16	-	-	-	-	-	-	-	-
17	-	-	-	-	-	-	-	-
18	-	-	-	-	-	-	-	-
19	-	-	-	-	-	-	-	-
20	-	-	-	-	-	-	-	-
21	-	-	-	-	-	-	-	-
22	-	-	-	-	-	-	-	-
23	P	P	-	-	P	P	-	-
24	-	-	-	-	-	-	-	-
25	-	-	-	-	-	-	-	-
26	-	-	-	-	-	-	-	-
27	-	-	-	-	-	-	-	-
28	-	-	-	-	-	-	-	-
29	-	-	-	-	-	-	-	-
30	-	-	-	-	-	-	-	-
31	-	-	-	-	-	-	-	-
32	-	-	-	-	-	-	-	-
33	-	-	-	-	-	-	-	-
34	-	-	-	-	-	-	-	-
35	-	-	-	-	-	-	-	-
36	-	-	-	-	-	-	-	-
37	-	-	-	-	-	-	-	-
38	-	-	-	-	-	-	-	-
39	-	-	-	-	-	-	-	-
40	-	-	-	-	-	-	-	-
41	P	P	P	P	-	-	-	-
42	-	-	-	-	-	-	-	-
43	-	-	-	-	-	-	-	-
44	-	-	-	-	-	-	-	-
45	-	-	-	-	P	P	P	P
46	-	-	-	-	-	-	-	-
47	-	-	-	-	-	-	-	-
48	-	-	-	-	-	-	-	-
49	-	-	-	-	-	-	-	-
50	-	-	-	-	-	-	-	-
51	-	-	-	-	-	-	-	-

52	-	-	-	-	-	-	-	-
53	-	-	-	-	-	-	-	-
54	-	-	-	-	-	-	-	-
55	-	-	-	-	-	-	-	-
56	P	P	-	-	P	P	-	-
57	-	-	-	-	-	-	-	-
58	-	-	-	-	-	-	-	-
59	-	-	-	-	-	-	-	-
60	-	-	-	-	-	-	-	-

Note: ‘-‘= negative, P = positive
